# Sentinel node biopsy as an adjunct to limb salvage surgery for epithelioid sarcoma of the hand

**DOI:** 10.1186/1477-7819-3-41

**Published:** 2005-06-29

**Authors:** Alex Seal, Raymond Tse, Bret Wehrli, Alex Hammond, Claire LF Temple

**Affiliations:** 1 University of British Columbia, Vancouver, British Columbia, Canada; 2 Division of Plastic Surgery, University of Western Ontario, London, Ontario, Canada; 3 Department of Pathology, University of Western Ontario, London, Ontario, Canada; 4 Department of Radiation Oncology, London Region Cancer Centre, London, Ontario, Canada

## Abstract

**Background:**

Epithelioid sarcomas of the hand are rare, high-grade tumors with a propensity for regional lymphatic spread approaching 40%.

**Case presentation:**

A 54-year-old male with an epithelioid sarcoma of the palm was treated with neoadjuvant radiation, wide excision, and two-stage reconstruction. Sentinel lymph node biopsy was used to stage the patient's axilla. Sentinel node biopsy results were negative. The patient has remained free of local, regional and distant disease for the follow-up time of 16 months.

**Conclusion:**

The rarity of this tumor makes definitive conclusions difficult but SLN biopsy appears to be a useful adjunct in the treatment of these sarcomas.

## Background

Epithelioid sarcoma is a rare, high-grade, soft tissue sarcoma. These tumors typically present on the extremities, in males who are 20 to 30 years of age. Overall 5 and 10-year survival rates are 70% and 42% respectively [1]. Epithelioid sarcoma is among a group of sarcomas with a propensity for regional lymphatic spread, with lymph node metastasis rates reported between 17%-80% [2-7]. Due to the risk of regional spread, sentinel lymph node biopsy (SLN) may be useful in the management of this tumor.

The success of SLN biopsy is based on the principle that the primary tumor drains to one or a few lymph nodes in the regional basin. Histopathological analysis of these sentinel nodes has been shown to reflect the histology of the entire lymphatic basin [8,9]. This approach is currently the least invasive and most accurate nodal staging procedure for breast cancer and melanoma [10,11]. However, SLN biopsy has not been thoroughly investigated for sarcoma.

We report a case of SLN biopsy in conjunction with limb salvage surgery and complex soft tissue, neurovascular, and staged tendon reconstruction for the management of an epithelioid sarcoma of the hand.

## Case presentation

**Figure 1:**
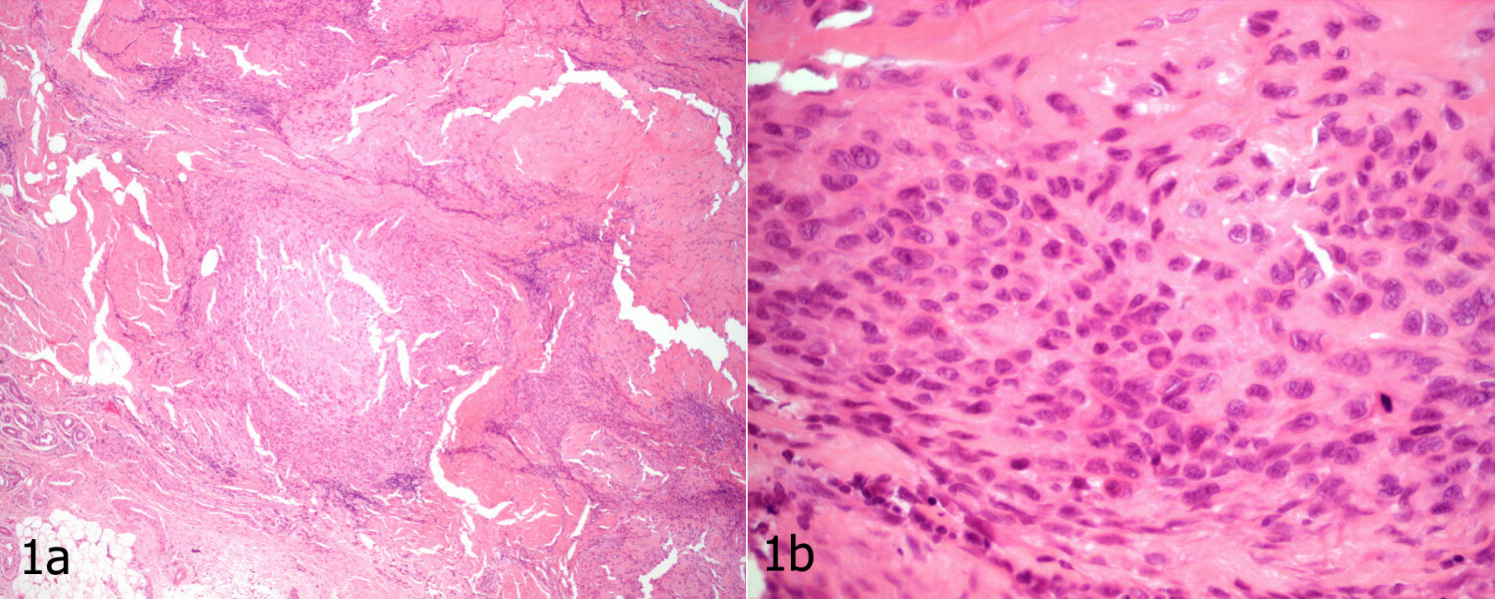
Epithelioid sarcoma a) Conglomerate of tumor nodules with central necrosis mimicking a necrotizing granulomatous process at low magnification (hematoxylin and eosin ×4) b) Epithelioid cells with abundant eosinophilic cytoplasm and prominent nuclear atypia, appreciated at high magnification (hematoxylin and eosin ×40), distinguishes epithelioid sarcoma from its benign mimics. Note the presence of a mitotic figure.

An otherwise healthy 54-year-old right-handed laborer presented with an eight-year history of a slowly enlarging "callus" in the palm of his right hand. He underwent excision at an outside institution. The operative note suggested that the tumor had the appearance of a sebaceous cyst with a large amount of surrounding tissue reaction. The neurovascular bundles were identified and preserved to the 4^th ^web space. An epithelioid sarcoma was identified upon histological analysis. Review of surgical pathology at our institution confirmed this diagnosis (Figure 1a and 1b). The excision was incomplete with tumor extending to several margins.

On examination, the patient had a transverse scar just proximal to the 4^th ^web space, with no palpable tumor. Neurovascular exam was normal, with normal range of motion of the associated digits. There were several small, palpable ipsilateral axillary nodes.

MRI of the right hand showed an ill-defined signal change within the palmar subcutaneous fat just deep to the surgical incision and distal to the 4^th ^and 5^th ^metacarpal-phalangeal joints, consistent with post-surgical changes and scarring. No discrete soft tissue mass was seen to suggest gross tumor. Edema signal was seen in the distal aspect of the lumbrical muscle between the ring and small flexor tendons. The interosseous muscles appeared uninvolved. No osseous or articular abnormalities were identified.

Preoperative computerized tomography (CT) of the chest was negative. The axilla was reported as having benign appearing lymph nodes, fatty in nature with no evidence of necrosis.

The patient received preoperative radiation of 50 Gy in 25 fractions. CT simulation was used for planning the gross tumor volume (GTV) to ascertain the depth to be treated using electrons. A customized lead cutout was designed to avoid treating the full width of the hand and to avoid normal tissues ulnarly, radially and at depth. Bolus was placed over the palm to ensure adequate superficial skin and scar dose whilst ensuring the dose at depth covered the tumor and previous operative bed. A daily dose of 2 Gy was delivered to a total of 50 Gy over a five week period. Moderate erythema of the palm occurred which healed well post-treatment. There was no significant edema post-irradiation. Dysethesias were reported on the radial aspect of the small finger; however, two point discrimination remained normal.

**Figure 2:**
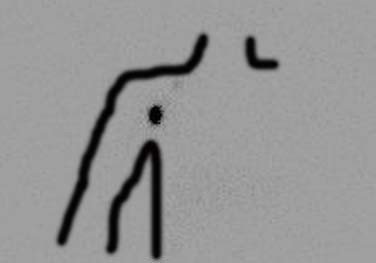
Preoperative lymphoscintigraphy identifies uptake of the radiolabelled tracer in a single axillary lymph node.

Surgery was performed 6 weeks following completion of radiotherapy. Preoperative lymphoscintography (figure 2) identified a single "hot" ipsilateral axillary lymph node, which was successfully removed. Vital blue dye was not used, as it was felt that the blue stained tissues would worsen visibility for the wide excision.

**Figure 3:**
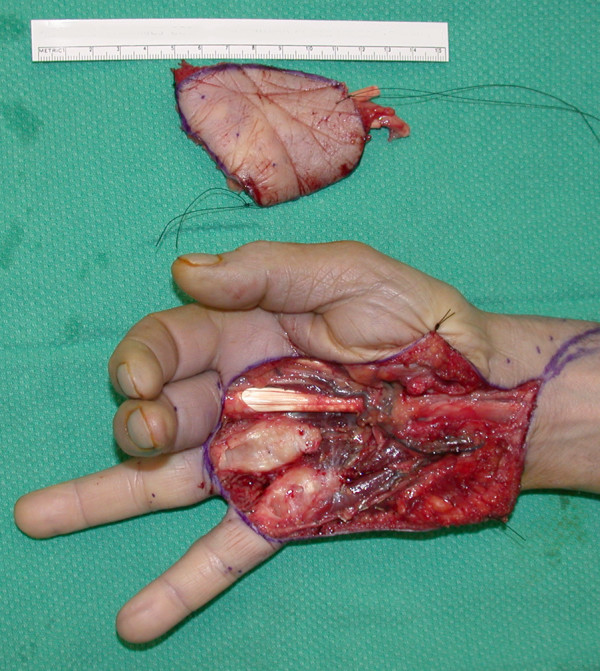
The appearance of the hand is shown following wide excision of skin, palmar fascia, flexor tendons, lumbricals, and neurovascular bundles. The small and ring fingers are postured in extension due to the absence of flexor tendons. The resection specimen is shown above.

Wide, *en bloc *excision was carried out including palmar skin, subcutaneous fat, palmar fascia, flexor digitorum superficialis and profundus tendons (including their associated lumbricals) to the small and ring finger, and the neurovascular bundles to the 3^rd ^and 4^th ^web space. The excision plane was carried down to the level of the fascia of the interossei, along the volar shafts of the metacarpals (Figure 3).

**Figure 4:**
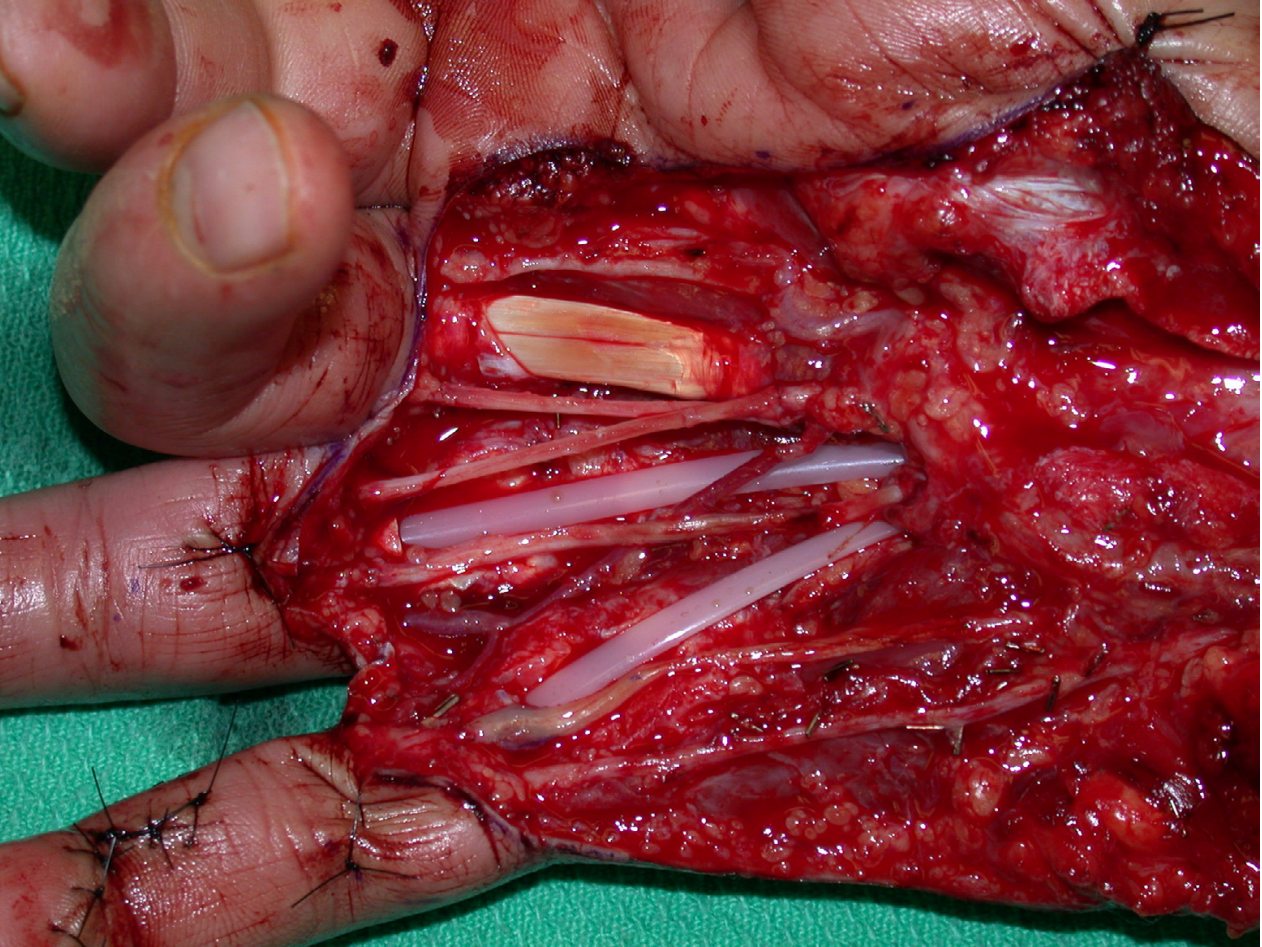
Sural nerve grafts, reversed Y-vein graft for digital revascularization of the small and ring fingers, and first-stage tendon reconstruction with silicone rod insertion is seen here. The construct was then covered with a contralateral free radial forearm flap.

Reconstruction included the placement of silicone rods to the small and ring fingers for the first part of a 2-stage flexor tendon reconstruction (Figure 4). Sural nerve grafts were placed to the small, ring and long finger. The small and ring fingers were revascularized from the superficial palmar arch with a Y-shaped vein graft harvested from the volar forearm.

The wound was then covered with a contralateral free radial forearm flap anastomosed to the radial artery and vena comitantes. The flap was innervated by neurorrhaphy of the lateral antebrachial cutaneous nerve in the flap to the palmar cutaneous branch of the median nerve. A small skin graft was used to cover the proximal pedicle to avoid compression.

Surgical pathology of the *en bloc *excision was negative for residual malignancy. Metastatic tumor was not identified within the sentinel lymph node following examination of multiple tissue levels of the node using both standard hematoxylin-eosin staining and immunohistochemical staining with antibodies against multiple cytokeratins and CD34, immunomarkers (frequently positive in epithelioid sarcoma).

**Figure 5:**
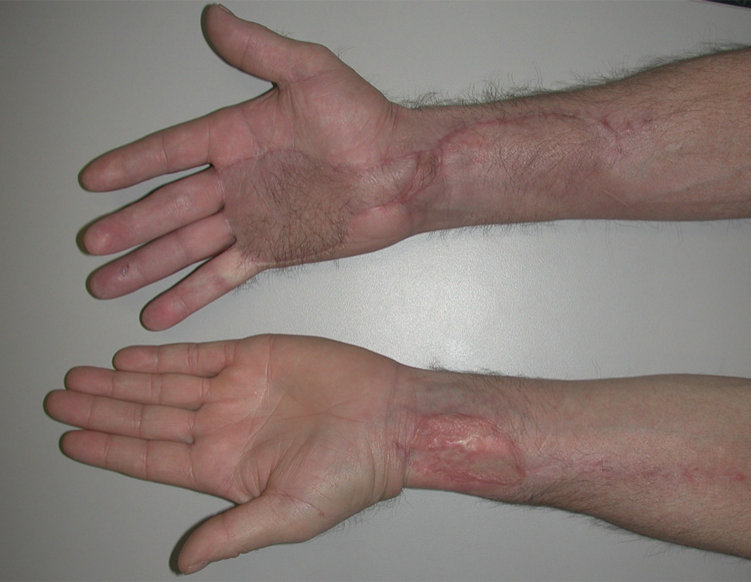
Prior to second-stage tendon grafting, the patient had a well-healed radial forearm flap on his right palm. The donor site on the left forearm was satisfactory.

At six months post-procedure, the radial forearm flap and donor site were well healed (Figure 5). The second-stage tendon reconstruction was undertaken by exchanging the silicone rods with extensor digitorum longus grafts from his 3^rd ^and 4^th ^toes for restoration of active finger flexion.

**Figure 6:**
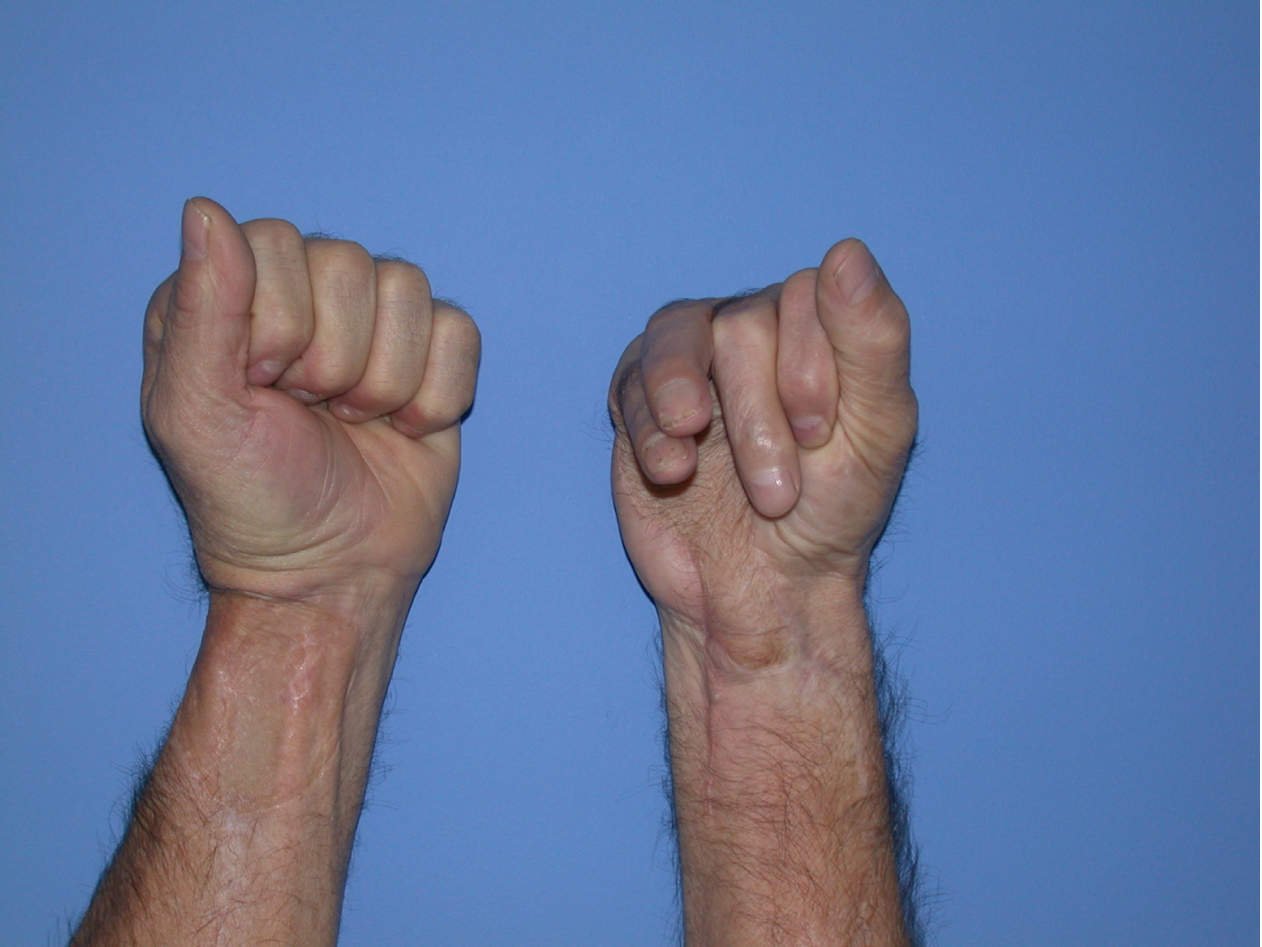
The final appearance of both hands after staged tendon grafting to the right ring and small fingers. There is functional restoration of composite finger flexion.

At 16 months post treatment, the patient remains free of local, regional and distant disease. He has regained acceptable hand function, with small and ring finger individual joint range of motion of 90 degrees at the metacarpal-phalangeal joints, 30 degrees at the proximal interphalyngeal joints, and 30 degrees at the distal interphalangeal joints (Figure 6). His moving two-point discrimination ranges from 5 to 7 mm.

## Discussion

Soft tissue sarcomas generally have a low incidence of regional lymph node metastasis (3–10%) [2] and regional lymph node recurrence (4–10%) [12,13]. Standard treatment includes wide local excision with pre-or postoperative radiotherapy. Limb salvage surgery provides acceptable local control comparable to amputation, with no difference in survival. [13-20] Multivariate analysis has demonstrated that the presence of metastasis at presentation is the single most important risk factor for local recurrence. [4] This likely reflects the more aggressive biologic potential of tumors that metastasize early and are more likely to fail local treatment.

Treatment of sarcomas of the hand is particularly challenging due to the concentrated and intricate anatomy, which makes sparing of critical structures difficult. Furthermore, the majority of these tumors are extra-compartmental, violating multiple tissue planes. Microsurgical skill for complex vascular and neural repair is an integral part of the overall planning of these cases, since without sophisticated reconstruction, limb salvage for hand sarcomas is unlikely to be useful. Free flaps are commonly required to restore function as well as to facilitate primary healing. [14-18]

The propensity of epithelioid sarcoma for regional spread supports the role of minimally invasive regional node staging procedures for prognosis and treatment. SLN biopsy has dramatically changed the management of melanoma and breast cancer. It has been investigated in the mapping of other tumors including penile [21], lung [22], colon [23,24], upper GI tumors [25], gynecologic cancer [26,27], thyroid cancer [28,29], and squamous cell carcinoma of the head and neck [7,30,31].

Given the success of the technique in other malignancies, it seems reasonable to apply SLN biopsy to soft tissue sarcomas of the extremity [8]. The role of SLN biopsy has not been extensively investigated in the treatment of sarcoma. In fact, there is only a single published report on its use in a child with rhabdomyosarcoma [9]. With the improved survival advantage of radical lymphadenectomy for clinically evident lymph node metastases from sarcoma, [4] accurate early detection of micrometastases may be important. Furthermore, patients with a negative sentinel lymph node biopsy for micrometastasis would be spared the morbidity of formal lymphadenectomy.

Identification of the sentinel node in sarcomas is more challenging than in breast and melanoma patients. Although successful identification of sentinel nodes exceeds 95% when using both a vital blue dye and a nuclear tracer [32,33], we avoided blue dye because it stain tissues and obscure planes. For resection in the hand, it is paramount to maintain precise visibility and a dye-free and bloodless field. Furthermore, when neoadjuvant radiotherapy is used, radiation-associated scarring of lymphatics could alter the accuracy of lymphatic mapping. Therefore, despite SLN biopsy, close follow-up of regional nodal basins is required. This includes assessment with clinical examination as well as with imaging such as high-resolution ultrasound. Our particular patient required serial chest computed-tomography scans for follow-up of unrelated, non-specific lung nodules. This provided a concurrent, detailed, and serial assessment of the benign appearance of his operated axillary bed.

## Conclusion

We report a case of epithelioid sarcoma of the hand successfully managed with a multi-disciplinary approach including neoadjuvant radiation, sentinel node biopsy and wide surgical excision. The rarity of this tumor makes definitive conclusions difficult but SLN biopsy appears to be a useful adjunct in the treatment of these sarcomas.

## Competing interests

In the past five years we have not received reimbursements, fees, funding, or salary from an organization that may in any way gain or lose financially from the publication of this manuscript, either now or in the future. No such an organization financed this manuscript (including the article-processing charge).

• We do not hold any stocks or shares in an organization that may in any way gain or lose financially from the publication of this manuscript, either now or in the future.

• We do not hold nor are applying for any patents relating to the content of the manuscript. We have not received reimbursements, fees, funding, or salary from an organization that holds or has applied for patents relating to the content of the manuscript.

• We have no other financial competing interests.

We do not have any non-financial competing interests (political, personal, religious, academic, intellectual, commercial or any other) to declare in relation to this manuscript.

## Authors' contributions

**CT**: Primary surgeon; report conception, writing, preparation and revision of manuscript, response to reviewers' questions, submission of manuscript, photographs

**RT**: First assistant surgeon; literature review, chart review, data collection, writing and preparation of manuscript

**AS**: Second assistant surgeon; literature review, chart review, data collection, writing and preparation of manuscript

**AH**: Radiation oncologist; chart review, writing and preparation of manuscript

**BW**: Pathologist; writing, preparation and revision of manuscript, histologic slide review, photographs
